# Effect of Oregano Essential Oil and Aqueous Oregano Infusion Application on Microbiological Properties of Samarella (Tsamarella), a Traditional Meat Product of Cyprus

**DOI:** 10.3390/foods7040043

**Published:** 2018-03-21

**Authors:** Beyza Ulusoy, Canan Hecer, Doruk Kaynarca, Şifa Berkan

**Affiliations:** 1 Department of Food Hygiene and Technology, Faculty of Veterinary Medicine, Near East University, Nicosia 99138, Cyprus; hecer01@gmail.com (C.H.); dkaynarca@hotmail.com (D.K.); 2 Değirmenlik Municipality Veterinary Affairs Directorate, Nicosia 99100, Cyprus; sifaberkan@hotmail.com

**Keywords:** Cypriotmeat product, dry-cured meat, food safety, meat technology, microbiology

## Abstract

Different types of dried meat products manufactured by different drying and curing methods are very common and well-known with a long history all over the world. Samarella (tsamarella) is one of these products and is famous among traditionally produced meat products in Cypriot gastronomy. The aim of this study was to investigate the effect of oregano essential oil (OEO) and aqueous oregano infusion (AOI) applications on the microbiological properties of samarella. In order to carry out this study, traditional methods were followed for experimental production of samarella. As a result of this study, five percent OEO application was found to be more effective to reduce microbiological counts but this ratio of OEO application was not accepted by panelists. According to all microbiological results correlated with the sensorial scores, it is concluded that one percent OEO application can be used for samarella production as an alternative preservative method.

## 1. Introduction

Dried meat products are very common and well known with a long history especially in Middle Eastern and South Asian countries. The best known traditionally produced dried meat products are qwanta, biltong, kilishi, two different regionally different produced cecina (Spanish and Mexican), charque, pemmican, rougan, roupu, jirge, kaddid and pastrami [1]. The typical microflora is mostly made up of *Micrococcaceae*, lactic acid bacteria and moulds and yeasts [2]. The halotolerant character of *Micrococcaceae* makes them to be stable during the production process. Yeasts are usually found in high numbers, up to 10^6^ CFU/g (Colony Forming Unit/gram), in dry-cured meat products [2,3]. The most frequently species isolated from the surfaces of dry-cured meat products are the general *Aspergillus*, *Eurotium* and *Penicillium* [4,5]. In some cases, unwanted spoilage moulds that contaminated during manufacture can develop on the surface of these products and may negatively affect the appearance, odour, taste and nutritional value of the products [5]. 

Samarella (tsamarella-τσαμαρέλλα in Greek) which is called Cypriot pastrami is also a type of sun dried meat product. This traditional dry-cured meat product of Cyprus is made by de-boned sheep and mainly goats’ meat. Samarellais defined as a kind of pastrami/pastourma, which is unique to Cypriot culture, salted and dried in the sun for preservation [6,7]. Samarella was produced in the past in the foothills of the Trodos Mountains by the farmer people, especially in the areas of Paphos and Dillirga for the purpose of meat preservation, generally from the meat of mouflon [8]. Today it is produced from the meat of goat or sheep of mature age and is still dried in the sun by traditional methods. After drying, it is washed and sprinkled with dry oregano that is accepted as a natural preserving agent and at the same time gives unique flavour. Samarella, considered as an appetizer in food culture today, used to be consumed with tomatoes for breakfast and also added into some dishes such as dry beans, cracked wheat, etc. Samarella is also a very important Cypriot meat product that is under protection of The Slow Food Presidia project. The Presidium aims to promote samarella in local markets and to bring it to international attention. According to the accepted rules of samarella manufacturing of Presidium, only from the thigh of the animal should be used because it is the leanest, most highly valued cut and best provides the earthy, rustic flavour and smoothness they are looking for. The leg is butterflied and cut into strips, immersed in salt and oregano and left to dry in the sun [9].

Today’s consumers prefer safe, additive-free and natural food of high nutritional value. In order to obtain safe and natural products, new approaches are being tried in food processing technology. Natural products, especially essential oils (EOs), used in the manufacture of dry cured meat are considered safe and natural additives for food preservation [10]. In the EU, essential oils are considered safe food additives at concentrations <2 mg/kg body-weight/day [11,12]. The use of EOs have a good perspective of application because they improve both safety and self-life of foodstuffs, mainly in beef, chicken, lamb or rabbit fresh meat [13]. Usage of oregano EO in meats was found effective in inhibiting spoilage microorganisms [14,15]. Among EOs, oregano EO has been long used as a flavouring agent for meat. The presence of several components of oregano EO has important antioxidant and antimicrobial properties. Major components of oregano EO were carvacrol (77.6%) followed by *p*-cymene (5.14%), *trans*-β-caryophyllene (2.45%), linalool (2.44%), γ-terpinene (2.35%) and thymol (2.11%), while other compounds were present under two percent [16]. 

According to the literature survey that we conducted for samarella, we have seen that the information in the scientific literature related to microbiology of this product is very scarce and the microorganisms participating in the final quality of the product have not been fully investigated. The aim of the present study was to investigate the effect of oregano essential oil and aqueous oregano infusion applications on the microbiological properties of samarella. Oregano is a natural ingredient of samarella. This is a reason why we planned oregano applications in this study. On the other hand, the study aimed to obtain a strong aroma and antimicrobial effect with these applications.

## 2. Materials and Methods 

### 2.1. Preparation of Oregano Infusion and Oregano Essential Oil

*Oregano* spp. (*Origanum minutiflorum*, *Origanum vulgare* L., *Origanum onites* L., *Origanum majorana* L.) essential oils were purchased from the manufacturer Ecodab Organic Plant Oils (Antalya, Turkey). According to the description of the manufacturer, oregano essential oil was obtained by steam-distillation of the plant leaves. In order to provide the similar conditions of aqueous oregano infusion (AOI), essential oregano oil (OEO) was mixed with 500 mL sterile distilled water in the ratios of 1% and 5%. *Origanum syriacum* L. (syn. *Majorana syriaca*) leaves, which is an endemic plant from Northern Cyprus, was used to prepare AOI. The infusion method was modified from the method described by Aksoy et al. [17]. Aqueous infusion of oregano leaves was prepared by soaking 50 g of leaves in 500 mL sterile distilled water in sterile flask. The flask was kept for 30min at room temperature with occasional shaking. The contents of flasks were then filtered under aseptic conditions with sterile equipment.

### 2.2. Experimental Production of Samarella

**Figure 1:**
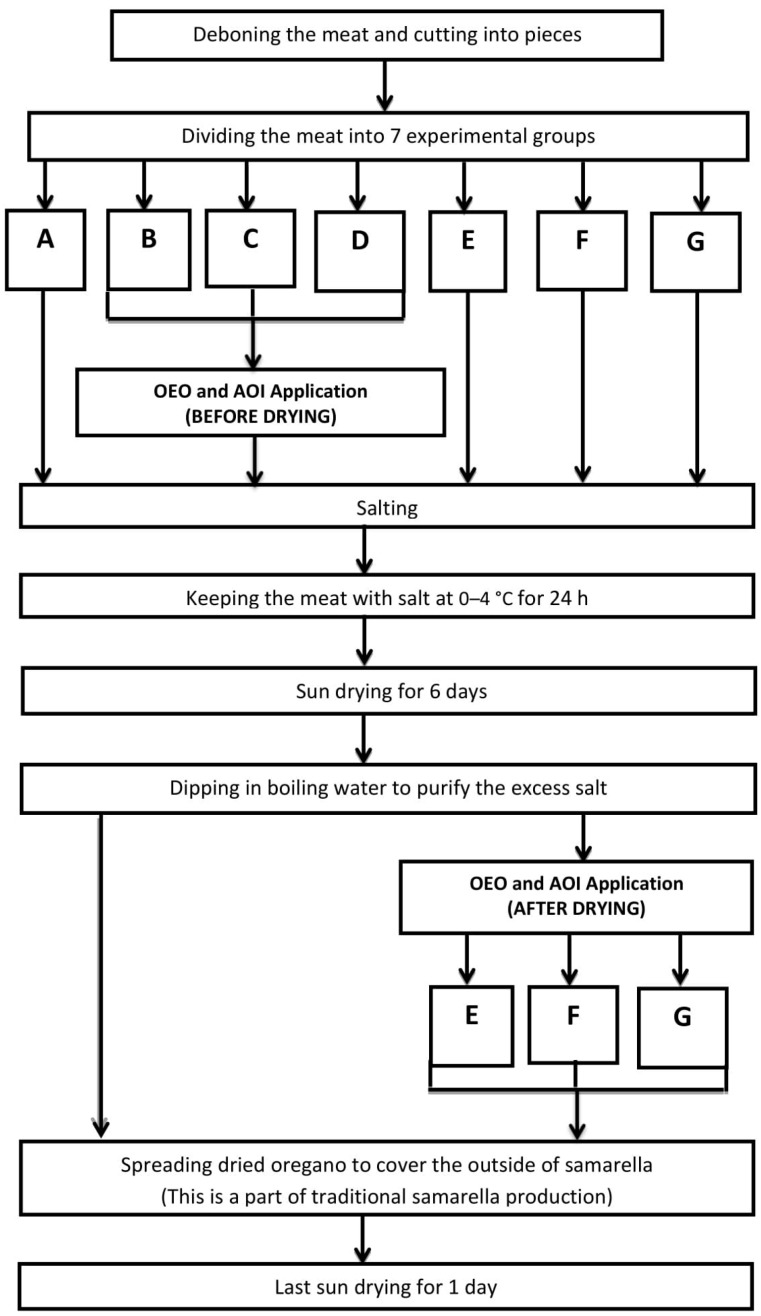
Production of samarella and experimental design. OEO: oregano essential oil; AOI: aqueous oregano infusion.

In order to carry out this study, the traditional method was followed for experimental production of samarella. All experimental samples were manufactured in the facility of a commercial samarella producer in Nicosia. Production was performed at the end of August, which is a suitable month for sun drying. Sheep meat was used for production. Two batches were produced on the same day and same parts of two sheep were used in experimental production. Seven experimental samples of samarella were hung up in an open cabinet surrounded with wire in order to protect from insects and left sun drying similar to traditional methods. The experiment was designed in duplicate. The flow diagram of samarella production and the experimental design is shown in Figure 1. OEO and AOI applications were done before and after the drying stage of samarella production. According to this experiment plan, the trial samples were coded as given in Table 1. The divided meats were put into a sterile sample bags with OEO and AOI, then bags were closed tightly and all were shaken gently for 10-min. In this way, samples were allowed to come into contact with prepared liquids.

### 2.3. Microbiological Analysis

Under aseptic conditions, 10 g of each experimental samarella sample was suspended and blended for 10 min into sterile 90 mL maximum recovery diluent. The suspensions were serially diluted up to appropriate ratio of dilution and plated onto selective media. After inoculation the samples on petri dishes, De Man, Rogosa, Sharpe (MRS) and Violet Red Bile Glucose (VRBG) agar were covered with a second layer of the same culture media before they were left to incubation. Baird Parker Agar Base supplied with an egg yolk tellurite emulsion. After incubation, the plates containing 300 ± 30 colonies were counted. Microbiological data were transformed into logarithms of the number and presented as colony forming unit CFU/g. The list of selective media and the incubation conditions are given in Table 2.

### 2.4. Physicochemical Analysis

Experimental samarella samples were analysed for their moisture content according to Association of Analytical Communities (AOAC) methods [18]. For pH measurements, 5 g of samples were homogenized with 45 mL distilled water in a blender for 1min and left to stand for 30min before measurement with a pH meter (WTW InoLab pH 7110, Berlin, Germany) calibrated with pH 7.0 and 4.0 buffers stored at room temperature (22 ± 2 °C). Mohr method was used to determine the salt content of samples described by Hecer and Ulusoy [19].

### 2.5. Sensorial Analysis

The panel consisting of 22 scientists between the ages of 25 and 65 from Near East Veterinary Faculty evaluated the product. The members of the panel previously knew and had tasted the traditional product samarella. The panelists were informed about the experimental groups and how to score the samples before they started. However, they did not know what the codes of the groups refer to. They asked to score external and internal appearance, texture, taste, odour and general acceptance of seven experimental samarella samples. Samples were coded with capital letters from A to G. Each attribute was rated on a five-point scale, with a score 1 equivalents to the lowest intensity of the attribute and the score 5 to the highest intensity of the attribute. Panelists were asked to drink water and eat plain white bread before tasting each sample in sensory sessions.

### 2.6. Statistical Analysis

All descriptive statistics of the search variables were calculated. For categorical variables frequency and percentage were calculated while the continuous variables were represented as arithmetic mean and standard error of mean (±SEM) and minimum–maximum values. Since the data did not meet parametric assumptions, non-parametric hypothesis tests were applied. Multiple groups were compared with a Kruskal–Wallis test and in case of significance a Mann–Whitney U test with Bonferroni correction was performed to test pairwise differences. SPSS (Demo Version 22.0, IBM, Armonk, NY, USA) software was used for all analyzes. The level of significance was accepted to be 0.05. Data was shown as the means (±SEM) of duplicate sets of experiments with three results for each analysis.

## 3. Results

According to the results of pH analyses, Sample B (6.08 ± 0.02) was found to have the highest and Sample C (5.90 ± 0.04) had the lowest pH mean value. When we compared the dry-matter percentages of the samples with control, we obtained that five percent OEO application after sun drying had the highest effect on drying. When one percent OEO was applied before sun drying stage (Sample C), lower dry-matter percentage was obtained compared to control. Salt percentages of all trial samples were obtained lower than control, although same amount of salt was added to all samples during manufacturing. Both before and after sun drying AOI samples (Samples D and G) had lower salt percentage than OEO samples (Samples B, C, E, F) and control group (Sample A). The results of all physicochemical measurements of our experiment are presented in Table 3.

In the current study, *Enterobacteriaceae* and anaerobic sulphite-reducing *Clostridia* counts were under the detectable value of 1 log CFU/g in all samples. Micrococcaceae counts decreased under to detectable value of 1 log CFU/g in five percent OEO applied samples (Samples B and E). Except for the AOI applied sample before drying (Sample D), in all samples, it was observed that Micrococcaceae counts decreased when compared to control group. A slight increase of Micrococcaceae was observed for Sample D. Micrococcaceae mean counts ranged between 2.16 ± 0.16 log CFU/g (Sample F) and 2.58 ± 0.01 log CFU/g (Sample D). Similarly, total aerobic mesophilic bacteria (TAMB) counts in five percent OEO applied samples (Samples B and E) decreased much more than in other experimental samples. TAMB counts ranged between 2.95 ± 0.01 log CFU/g (Sample E) and 3.85 ± 0.03 log CFU/g (Sample A). Statistical analyses indicated that both five percent and one percent OEO applications caused significant difference in TAMB counts. As seen in Table 4, AOI and OEO did not fully decontaminate lactic acid bacteria (LAB) on samarella but a significantly decrease was obtained in OEO implicated samples (Samples B, C, E and F) compared to control group. LAB mean counts ranged between 2.17 ± 0.03 log CFU/g (Sample E) and 2.69 ± 0.01 log CFU/g (Sample A). According to the evaluation of mold counts it was detected that only five percent OEO application after sun drying caused adecrease inmould counts under the detectable value of 1 log CFU/g. In all OEO applied samples much more reduction in mould counts was observed compared to AOI applied samples. When starter cultures are not used, undesired mould genera can grow on meat products. The mean values of microbiological analysis results were presented in Table 4.

According to mean scores of all attributes for each sample, one percent OEO before drying and AOI after drying (Samples C and G) had the highest scores and five percent OEO before drying (Sample B) had the lowest sensorial scores. Sample C had the highest scores for external appearance, internal appearance and general acceptance attributes and Sample G had the highest scores for texture and taste attributes. For odour evaluation, Sample G had the highest score with Sample A. Sample C (one percent OEO before sun drying) and Sample G (AOI after sun drying) get the highest mean scores and Sample B (five percent OEO before sun drying) get the lowest mean scores. As presented in Table 5, the scores of taste, odour and general acceptability were also lower than the scores for one percent OEO application samples. When we evaluated taste scores, Samples C, D and E achieved statistically different scores. 

## 4. Discussion

We observed that OEO or AOI applications did not create big differences in pH values (Table 3). These results are similar to results of the study performed by Petrou et al. [20]. They concluded that chitosan dipping or oregano oil treatments to chicken breast meat had no significant effect on pH values. Govaris et al. [14] also reported no effect of oregano oil on pH measurements of minced sheep meat during refrigerated storage. Fernandes et al. [21] also found no effect of oregano extract on pH of sheep burgers as similar as we found in our study. As presented in Table 3, dry-matter percentages of five percent OEO applied samples (Samples B and E) were higher than one percent OEO applied samples (Samples C and F). According to these dry matter analysis results, it can be concluded that application of OEO after sun drying may be more effective on drying when the ratio of OEO is higher. Dipping the meats in AOI helped reducing the excess salt of samarella. Because of that, Samples D and G had lower salt percentage. In this study, pH and dry matter analysis results of all experimental applications showed us that OEO and AOI applications to samarella did not negatively affect the production.

The typical microflora of dry-cured meat products consists of *Micrococcaceae*, lactic acid bacteria, moulds and yeasts [2]. On the other hand, several Gram positive bacteria (lactic acid bacteria, *Brochothrix thermosphacta*, *Clostridia*) are reported to be spoilage bacteria for meat products; however, some researchers concluded that some of them give the products characteristic properties [22,23]. In our study, applying five percent OEO seems to be inhibitive for *Micrococcaceae* counts and caused reduction in TAMB counts nearly 1 log unit. As reported by Pathokas et al. [22], a large number of LAB general and species have been known to be responsible for meat spoilage. LAB are generally resistant to the most of the preservation methods. This situation increases the importance of methods for reducing the number of LAB under acceptable levels. We observed reduction in LAB counts for all experimental sample groups compared to control group. LAB counts in five percent OEO applied sample groups before and after drying (Samples B and E) decreased much more than other groups. Further studies should be performed on taxonomic data of LAB on samarella in order to understand if they are a part of normal samarella microflora and to understand whether or not they are spoilage bacteria.

As International Commission on Microbiological Specifications for Foods (ICMSF) [24] reported, raw cured or salted meats have had a very good record of safety. Although some of them have been implicated in outbreaks of food-borne illness, particularly botulism, the incidents have usually involved home-prepared products. In view of this, we evaluated the effect of OEO and AOI on undesired microorganisms. In a recent study, it was concluded that carvacrol and thymol can be a good alternative as natural antimicrobial agents in meat products against meat spoilage caused by Gram negative bacteria (*Pseudomonads*, *Enterobacteriaceae*, *Shewanella putrefaciens*) [22,23]. This data supports the absence of *Enterobacteriaceae* in our study. 

Growth of xerotolerant and xerophilic moulds which come from environmental conditions is very common. Species from the genera *Aspergillus*, *Eurotium* and *Penicillium* are most frequently isolated molds from dry-cured meat products [4,5,25]. In our study, OEO seemed to be effective for reducing the counts of moulds. As we mentioned for other microbiological counts, similarly five percent of OEO applications (Samples B and E) were obtained more effective than one percent OEO applications (Samples C and F) for mould counts. AOI application before and after sun drying was not effective to reduce mould counts compared to control (Sample A).

The acceptance and admiration of sensorial attributes of dry-cured products by consumers are very important in order to give a decision for new applications to foods. We evaluated the sensorial scores with this perspective. Although five percent OEO application caused a stronger effect on microbiological parameters, it was not liked by the panelists. This may be due to the intense odour of five percent OEO.

As the conclusion of this study, five percent OEO application was more effective to reduce microbiological counts but this ratio of OEO application was not accepted by panelists. Panelists liked one percent OEO before sun drying (Sample C) and AOI after sun drying (Sample G). When we correlate these results with microbiological results one percent OEO application before sun drying can be accepted as a good alternative preservative method for samarella as well as providing a good and natural aroma to the product. Further microbiological quality survey studiesof this Cypriot meat product should be performed and microbiological criteria need to be applied to this traditional meat product. On the other hand, new studies should be planned for identification normal microflora of samarella. For example, LAB are reported as part of dry meats’ normal microflora, but the spoilage character of some LAB taxa is unclear. In our study, although OEO had good ability to reduce LAB counts, it did not inhibit LAB completely. This can be evaluated as a desired result of the study because LAB help the product gain its characteristics properties.

## Figures and Tables

**Table 1:** Sample codes of the study. OEO: oregano essential oil; AOI: aqueous oregano infusion.

Group of Samples	Application
SAMPLE A	No Application
SAMPLE B	Immersion to 5% OEO before sun drying stage
SAMPLE C	Immersion to 1% OEO before sun drying stage
SAMPLE D	Immersion to AOI before sun drying stage
SAMPLE E	Immersion to 5% OEO after sun drying stage
SAMPLE F	Immersion to 1% OEO after sun drying stage
SAMPLE G	Immersion to AOI after sun drying stage

**Table 2:** Selective media and incubation conditions.

Analysed Microorganisms	Selective Medium	Incubation Parameters
*Micrococcaceae*	Baird Parker Agar (BPA)	37 °C/48 h
*Enterobacteriaceae*	Violet Red Bile Glucose (VRBG) Agar	37 °C/24 h
Moulds	Sabouraud Dextrose Agar (SDA)	25 °C/72–96 h
Total aerobic mesophilic bacteria (TAMB)	Standard Plate Count Agar (PCA)	37 °C/48 h
Anaerobic sulphite-reducing *Clostridia*	Sulfite Polymyxin Sulfadiazine (SPS) Agar	37 °C/48 h
Lactic acid bacteria (LAB)	De Man, Rogosa, Sharpe (MRS) Agar	30 °C/48–72 h

**Table 3:** Physicochemical analysis results.

	pH		Dry Matter (%)		Salt (%)	
	Mean ± SEM	Min–Max	Mean ± SEM	Min–Max	Mean ± SEM	Min–Max
Sample A	5.98 ± 0.04	5.90–6.10	65.70 ± 0.07	65.50–65.20	17.60 ± 0.02	17.50–17.70
Sample B	6.08 ± 0.02	6.00–6.10	65.17 ± 0.02 ^a^	65.10–65.20	14.00 ± 0.02 ^a^	13.90–14.10
Sample C	5.90 ± 0.04 ^b^	5.80–6.00	54.72 ± 0.06 ^a,b^	54.50–54.90	12.90 ± 0.03 ^a,b^	12.80–13.00
Sample D	5.98 ± 0.03 ^b^	5.90–6.10	66.75 ± 0.07 ^a,b,c^	66.50–67.00	8.83 ± 0.05 ^a,b,c^	8.70–9.00
Sample E	5.95 ± 0.02 ^b^	5.90–6.00	69.08 ± 0.40 ^a,b,c,d^	69.00–69.20	14.58 ± 0.30 ^a,b,c,d^	14.50–14.70
Sample F	5.98 ± 0.01	5.90–6.00	64.43 ± 0.42 ^a,b,c,d,e^	64.30–64.60	13.52 ± 0.47 ^a,b,c,d,e^	13.30–13.60
Sample G	5.93 ± 0.04	5.80–6.10	68.43 ± 0.42 ^a,b,c,d,e,f^	68.30–68.60	8.87 ± 0.03 ^a,b,c,d,e,f^	8.80–9.00

Each letter symbolizes statistically significant difference from a specific sample. (*p* ≤ 0.05); ^a^ Significant difference from Sample A; ^b^ Significant difference from Sample B; ^c^ Significant difference from Sample C; ^d^ Significant difference from Sample D; ^e^ Significant difference from Sample E; ^f^ Significant difference from Sample F; Data are the means (±SEM) of duplicate sets of experiments with three results for each analysis, SEM: Standard error of the mean.

**Table 4:** Microbiological analysis results (log CFU/g).

	*Micrococcaceae*		TAMB		LAB		Moulds	
	Mean ± SEM	Min–Max	Mean ± SEM	Min–Max	Mean ± SEM	Min–Max	Mean ± SEM	Min–Max
Sample A	2.47 ± 0.01	2.46–2.48	3.85 ± 0.03	3.84–3.86	2.69 ± 0.01	2.69–2.70	3.69 ± 0.01	3.69–3.70
Sample B	udl *	--	2.99 ± 0.01 ^a^	2.99–3.00	2.48 ± 0.01 ^a^	2.46–2.48	1.99 ± 0.01 ^a^	1.98–2.00
Sample C	2.32 ± 0.01 ^a^	2.31–2.33	3.32 ± 0.01 ^a,b^	3.31–3.32	2.60 ± 0.01 ^a,b^	2.69–2.70	1.99 ± 0.01 ^a^	1.97–2.01
Sample D	2.58 ± 0.01 ^a,c^	2.57–2.58	3.52 ± 0.01	3.51–3.53	2.62 ± 0.01 ^a,b,c^	2.61–2.62	3.40 ± 0.07 ^a,b,c^	3.05–3.47
Sample E	udl *	--	2.95 ± 0.01 ^a,b,c,d^	2.94–2.95	2.17 ± 0.03 ^a,b,c,d^	2.16–2.18	udl *	--
Sample F	2.16 ± 0.16	1.99–2.98	3.17 ± 0.16 ^a,d,e^	3.00–3.99	2.60 ± 0.01 ^a,b,d,e^	2.59–2.60	2.25 ± 0.01 ^a,b,c,d^	2.24–2.26
Sample G	2.30 ± 0.01 ^c,d,f^	2.29–2.30	3.39 ± 0.01	3.39–3.41	2.62 ± 0.01	2.61–2.63	3.55 ± 0.10	3.53–3.60

Each letter symbolizes statistically significant difference from a specific sample. (*p* ≤ 0.05); * udl: Under detectable limit of 1 log CFU/g, CFU: Colony forming unit; Data are the means (±SEM) of duplicate sets of experiments with three results for each analysis, SEM: Standard error of the mean; TAMB: Total aerobic mesophilic bacteria. LAB: Lactic acid bacteria; ^a^ Significant difference from Sample A; ^b^ Significant difference from Sample B; ^c^ Significant difference from Sample C; ^d^ Significant difference from Sample D; ^e^ Significant difference from Sample E; ^f^ Significant difference from Sample F.

**Table 5:** Panelists’ sensorial scores.

	External		Internal		Texture		Taste		Odour		General Acceptance	
	Mean ± SEM	Min–Max	Mean ± SEM	Min–Max	Mean ± SEM	Min–Max	Mean ± SEM	Min–Max	Mean ± SEM	Min–Max	Mean ± SEM	Min–Max
Sample A	3.77 ± 0.16	2.00–5.00	3.86 ± 0.15	2.00–5.00	3.59 ± 0.10	3.00–4.00	3.23 ± 0.09	3.00–4.00	3.77 ± 0.11	3.00-5.00	3.50 ± 0.12	3.00–5.00
Sample B	2.77 ± 0.14	2.00–4.00	2.73 ± 0.11	2.00–4.00	2.14 ± 0.07	2.00–3.00	1.86 ± 0.17	1.00–4.00	2.18 ± 0.10	1.00–3.00	2.14 ± 0.07	2.00–3.00
Sample C	4.14 ± 0.11	3.00–5.00	4.14 ± 0.07	4.00–5.00	4.00 ± 0.06 ^a^	3.00–5.00	4.09 ± 0.13 ^a^	3.00–5.00	3.59 ± 0.10	3.00–4.00	4.09 ± 0.11 ^a^	3.00–5.00
Sample D	3.18 ± 0.08 ^a,b^	3.00–4.00	3.23 ± 0.11 ^a,b^	2.00–4.00	2.23 ± 0.09 ^a^	2.00–3.00	2.18 ± 0.08 ^a^	2.00–3.00	2.45 ± 0.12 ^a^	2.00–4.00	2.14 ± 0.07 ^a^	2.00–3.00
Sample E	3.86 ± 0.11	3.00–5.00	3.45 ± 0.10 ^a^	3.00–4.00	2.64 ± 0.12 ^a,b^	1.00–3.00	2.23 ± 0.11 ^a^	1.00–3.00	2.50 ± 0.12 ^a,b^	1.00–3.00	2.50 ± 0.14 ^a,b,d^	1.00–4.00
Sample F	3.73 ± 0.13 ^c,d^	3.00–5.00	3.41 ± 0.14 ^a,b^	3.00–5.00	3.50 ± 0.15 ^c^	2.00–5.00	3.18 ± 0.12	2.00–4.00	3.09 ± 0.09 ^a,c,e^	2.00–4.00	3.09 ± 0.11 ^a,e^	2.00–4.00
Sample G	3.68 ± 0.12 ^c,d^	3.00–5.00	3.68 ± 0.13 ^c,d^	2.00–5.00	4.41 ± 0.15 ^c^	3.00–5.00	4.23 ± 0.11	3.00–5.00	3.77 ± 0.16 ^f^	3.00–5.00	3.80 ± 0.10 ^a^	3.00–5.00

Each letter symbolizes statistically significant difference from a specific sample. (*p* ≤ 0.05); ^a^ Significant difference from Sample A; ^b^ Significant difference from Sample B; ^c^ Significant difference from Sample C; ^d^ Significant difference from Sample D; ^e^ Significant difference from Sample E; ^f^ Significant difference from Sample F; Data are the means (±SEM) of duplicate sets of experiments with three results for each analysis, SEM: Standard error of the mean.
